# Is hyperuricemia an independent prognostic factor for IgA nephropathy: a systematic review and meta-analysis of observational cohort studies

**DOI:** 10.1080/0886022X.2021.2019589

**Published:** 2022-02-14

**Authors:** Kang Zhang, Long Tang, Shang-shang Jiang, Yue-fen Wang, Yuan Meng, Meng-di Wang, Fang-qiang Cui, Zhen Cai, Wen-jing Zhao

**Affiliations:** a Department of Nephrology, Beijing Hospital of Traditional Chinese Medicine, Capital Medical University, Beijing, China; b Dongzhimen Hospital Affiliated to Beijing University of Chinese Medicine, Beijing, China

**Keywords:** Immunoglobulin A nephropathy, prognosis, hyperuricemia, serum uric acid

## Abstract

**Background:**

Hyperuricemia has been reported to be correlated with IgA nephropathy (IgAN). However, whether hyperuricemia or elevated serum uric acid (SUA) is an independent prognostic factor of IgAN remains unknown. Therefore, this systematic review and meta-analysis evaluated the prognostic value of hyperuricemia and elevated SUA in IgAN.

**Methods:**

Databases including PubMed, EMBASE, the Cochrane Central Register of Controlled Trials (CENTRAL), and Open Gray were reviewed systematically. The kidney failure events of IgAN were defined as a doubling of serum creatinine, halving of eGFR, end-stage renal disease (ESRD), or death. The risk ratio (RR) between hyperuricemia and IgAN-caused kidney failure was evaluated before and after adjustment for relevant covariates. The RR between elevated SUA and IgAN-caused kidney failure was evaluated after adjustment for relevant covariates.

**Results:**

A total of 11 548 patients from 14 studies were included in this meta-analysis. Hyperuricemia was found to be an independent prognostic factor of IgAN (unadjusted RR = 2.79, 95% CI = 1.93–4.03, *p* for heterogeneity <0.00001, *I*^2^ = 91%; adjusted RR = 2.12, 95% CI = 1.64–2.73, *p* for heterogeneity = 0.86, *I*^2^ = 0%). Subgroup and sensitivity analyses confirmed the stability of these results. Similarly, elevated SUA was positively correlated with kidney failure events of IgAN (adjusted RR = 1.25, 95% CI = 1.19–1.31, *p* for heterogeneity = 0.6, *I*^2^ = 0%).

**Conclusion:**

Our meta-analysis showed that hyperuricemia and elevated SUA were both independently associated with an increased incidence of kidney failure events in IgAN patients.

## 1. Introduction

Immunoglobulin A nephropathy (IgAN), one of the most common forms of chronic glomerulonephritis, is also one of the most common causes of kidney failure worldwide [1]. Indeed, 10–30% of IgAN patients reached ESRD during the first 10 years after diagnosis [2,3]. Hence, early intervention and identification of novel prognostic factors of IgAN may decrease the risk of kidney failure.

Multiple studies have identified risk factors correlated with a poor prognosis of IgAN patients, namely hypertension, proteinuria, low estimated glomerular filtration rate (eGFR), high neutrophil-to-lymphocyte ratio, lower bilirubin and pathological injuries, such as the presence of crescents and the MEST score including mesangial hypercellularity (M), endocapillary hypercellularity (E), segmental glomerulosclerosis (S) and tubular atrophy/interstitial fibrosis (T) [4–8]. Hyperuricemia is an independent risk factor for segmental glomerulosclerosis and tubular atrophy/interstitial fibrosis [9], while glomerulosclerosis and renal tubular atrophy/interstitial fibrosis are independent prognostic factors in IgAN patients [10]. Therefore, it is reasonable to hypothesize that SUA level may be related to the prognosis of IgAN.

Previous meta-analyses have shown that hyperuricemia is associated with an increased risk for developing acute kidney injury (AKI) and chronic kidney disease (CKD) [11,12]. However, no meta-analysis has investigated the relationship between hyperuricemia and the prognosis of IgAN.

Here we have performed a systematic review and meta-analysis to investigate the prognostic value of hyperuricemia and elevated SUA in IgAN patients.

## 2. Methods

### 2.1. Search strategy

A systematic search of PubMed, the Cochrane Central Register of Controlled Trials (CENTRAL), and EMBASE (last update in May 2021) was conducted independently by two investigators. We also searched for gray literature in Open Gray. The following terms and their combinations were used for searching the databases: ‘hyperuricemia’, ‘uric acid’, ‘Glomerulonephritis, IGA’, ‘IGA nephropathy’, ‘Immunoglobulin A Nephropathy’, ‘Berger* disease’, ‘IGA Type Nephritis’. Only fully published studies in English were included. Corresponding references of each study were also scanned for relevant studies. All studies were carefully investigated to identify duplicate data. The protocol of this study was registered in the PROSPERO under the number CRD 42019118493.

### 2.2. Inclusion criteria

The following inclusion criteria were performed to select relevant studies: (1) participants (P), only adult patients (>18 years old) with biopsy-proven IgAN were included; (2) exposures (E), hyperuricemia as defined by the authors for each gender or an increase of 1 mg/dL in SUA levels; (3) comparisons (C), non-hyperuricemia; (4) outcomes (O), doubling of the serum creatinine level, halving of eGFR, death, or ESRD, ESRD was defined as eGFR < 15 mL/min/1.73 m^2^ or initiation of dialysis or transplantation; (5) study design (S), observational cohort studies; (6) sufficient information was provided to evaluate hazard ratio (HR) or RR and 95% confidence intervals (CIs). Case reports, letters, conference abstracts, reviews, and non-clinical studies were excluded due to the lack of sufficient data. To ensure the quality of our study, the process of this meta-analysis was conducted according to the criteria of the MOOSE checklist (Additional File 1).

### 2.3. Data extraction and qualitative assessment

The Newcastle Ottawa quality assessment scale (NOS) [13] was used to estimate the quality of the included studies. The study was defined as ‘high-quality’ when its NOS score was higher than six. Moreover, two investigators independently extracted the required information from all primary studies, including the name of the first author, year of publication, the number of patients included, sex, age, country, eGFR, proteinuria, medication use, follow-up data, and kidney failure outcomes.

### 2.4. Statistical analysis

The statistical analysis was conducted by the Quality of Reporting of Meta-Analysis guidelines and The Cochrane Library handbook [14,15]. The risk estimates were evaluated by different measures including HR using Cox proportional hazards regressions, or RR using logistic regressions. HR or RR from univariate and multivariate analyses were extracted from each study. If HR or RR were not reported in the studies, we used the original data to calculate the unadjusted HR or RR. HRs were considered the same as RRs. We calculated and pooled both the unadjusted and fully adjusted risk coefficients representing the correlation between hyperuricemia and kidney failure events in IgAN patients. For SUA levels, only the fully adjusted risk coefficients were calculated and pooled. Random-effect models were used to pool RRs or HRs. The heterogeneity of the studies was statistically assessed using the chi-squared and *I*^2^ tests [16]. Subgroup analysis was also performed to explore the heterogeneity of studies. Sensitivity analysis was conducted by removing one study at a time to assess the influence of a single study on the overall risk estimate. Funnel plots, Begg’s and Egger’s tests, as well as the trim-and-fill computation were used to evaluate publication bias. The STATA 15.1 and Review Manager 5.3 software were used for data analysis and graph formation.

## 3. Results

### 3.1. Characteristics of selected studies

**Figure 1.**
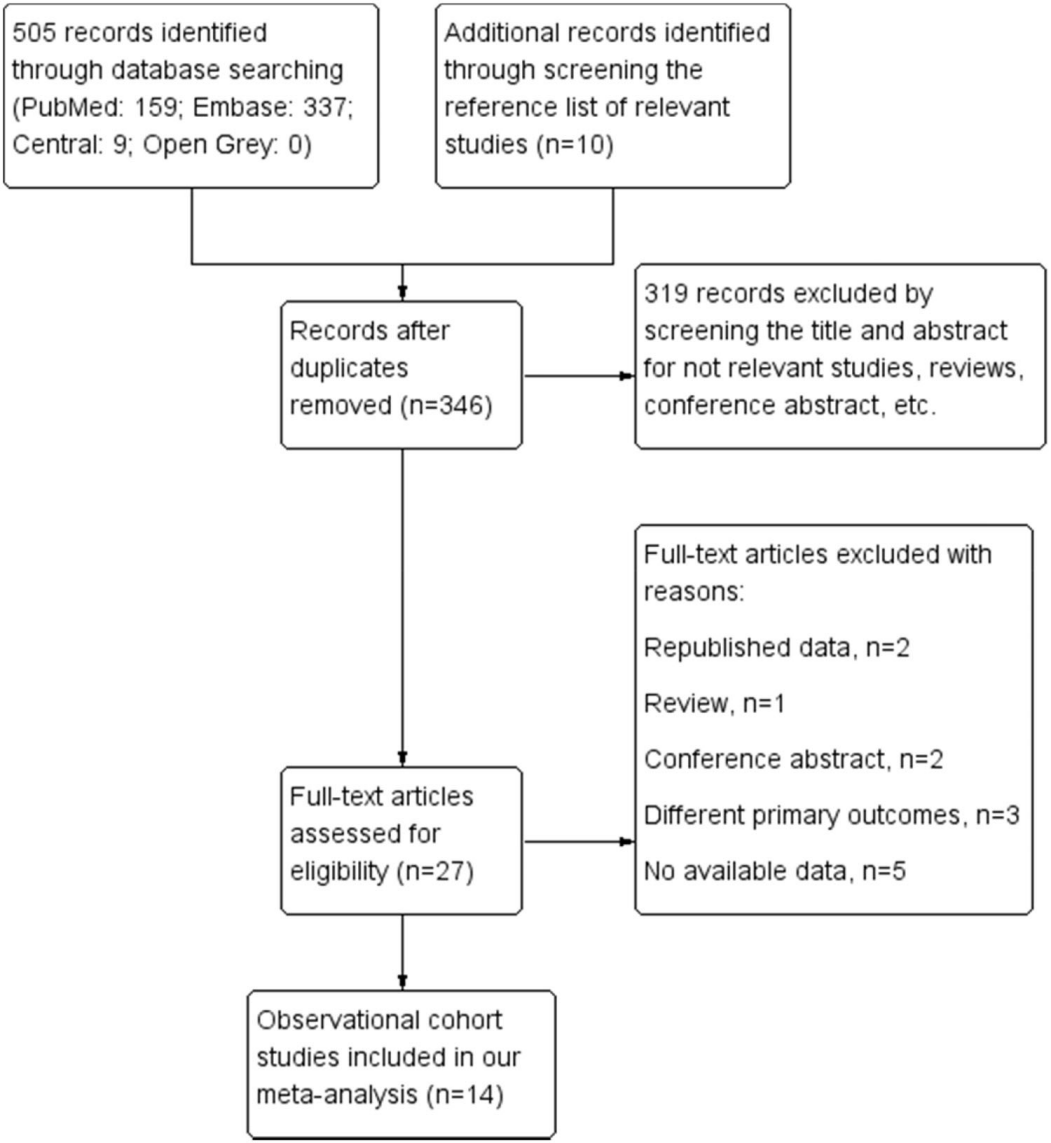
Flow diagram of study selection.

A total of 515 articles were identified using our search strategy, while 346 were considered potentially relevant after screening titles and abstracts and excluding duplicated articles. Among the 346 articles, 319 were excluded due to lack of data, studies were irrelevant or studies were not observational cohorts. Among the 27 articles, after a full-text review, 13 articles were excluded: two contained duplicated data, one was a review, two were conference abstracts, three measured irrelevant primary outcomes, and five lacked enough data for RR estimation. Finally, a total of 11 548 patients from 14 studies were included in this meta-analysis [17–30]. The literature screening process is shown in Figure 1.

Table 1 shows the characteristics of the fourteen studies that were included in our study. Twelve of the 14 studies were conducted in Asia (China, Japan, and Korea) [17–22,24–27,29,30], and two were conducted in Western countries (Turkey and Italy) [23,28]. Eleven of the fourteen studies estimated the association between hyperuricemia and kidney failure events in IgAN [18–24,26–29], in which RR was calculated using dichotomous variables. Only five studies estimated the relationship between elevated SUA and kidney failure events in IgAN [17,23,25,27,30], in which HR was calculated using continuous variables relative to 1 mg/dL. Thirteen of the 14 studies had a NOS score >6 [17,19–30], which indicates that most studies were of high quality (Additional File 2).

**Table 1.** Characteristics of included studies.

Author/year	Country	Study type	Follow-up^a^, (years)	Selective criteria for hyperuricemia (mg/dL)	Patients (*n*)	Age^a^, (years)	Men, *n* (%)	eGFR^a^ (ml/min/1.73 m^2^)	Proteinuria^a^ (g/day)	Events		URR (UHR) (95% Cl)
										HPU	Control	
Wen et al. [30] 2021	China	RCS	3.4	>7.06^b^ >6.05^c^	1096	32.67^b^ 34.65^c^	475 (43.3)	83.76^b^ 89.75^c^	1.90^b^ 1.64^c^	N.R.	N.R.	N.R.
Lu et al. [29] 2020	China	RCS	2.08	>7.06^b^ >6.05^c^	193	33.9	74 (38.34)	90.6	1.32	7/65	3/128	4.62 [1.19, 17.88]^&^
Russo et al. [28] 2020	Italy	RCS	6	>7.7^b^ >6.2^c^	145	47	102 (70)	45	2.6	18/48	20/97	1.82 [1.07, 3.10]^&^
Oh et al. [27] 2020	Korea	RCS	6.1	>6^b^ >5^c^	4339	39.3	2148 (49.5)	75.1	N.R.	N.R.	N.R.	1.38 [1.33, 1.43]^&^
Ruan et al. [21] 2018	China	RCS	2.6	>7.06^b^ >6.05^c^	206	33.2	86 (41.7)	88.4	N.R.	16/84	2/122	11.62 [2.74, 49.21]^&^
Liu et al. [19] 2018	China	PCS	3.7	N.R.	869	34	441 (50.7)	84.16	2.22	N.R.	N.R.	7.03 [4.13, 11.98]^&^
Matsukuma et al. [20] 2017	Japan	RCS	5.1	N.R.	826	35	372 (45)	81 (M group)^b^ 84 (M group)^c^ 64 (H group)^b^ 58 (H group)^c^	N.R.	90/435	27/391	2.30 [1.99, 4.50]*^&^
Caliskan et al. [23] 2016	Turkey	PCS	2.8	>7.0^b^ >6.5^c^	111	35	69 (62.2)	56	1.8	24/50	17/61	1.72 [1.05, 2.83]^&^
Li et al. [18] 2016	China	RCS	5.3	N.R.	1121	33.51	554 (49.4)	90.92	1.28	N.R.	N.R.	3.65 [2.6, 5.11]^&^
Moriyama et al. [25] 2014	Japan	RCS	7.9	N.R.	1012	33	410 (40.5)	78.5	1.19	N.R.	N.R.	1.57 [1.39, 1.77]^#^
Li et al. [17] 2014	China	PCS	3.1	N.R.	703	32	361 (51.4)	86.5	1.60	N.R.	N.R.	1.28 [1.17, 1.4]^#^
Cheng et al. [26] 2013	China	PCS	4.9	>7.06^b^ >6.0^c^	348	34.29	191 (54.9)	N.R.	N.R.	31/66	52/282	2.55 [1.79, 3.63]^&^
Shi et al. [22] 2012	China	RCS	5	>7.0^b^ >6.0^c^	353	35.0	138 (39.1)	N.R.	N.R.	31/112	19/241	3.51 [2.08, 5.94]^&^
Ohno et al. [24] 2001	Japan	RCS	10.4^b^ 9.9^c^	>7.0	226	50.1^b^ 47.5^c^	144 (63.7)	N.R.	1.05	27/48	50/156	1.76 [1.25, 2.46]^&^

Author/year	ARR(AHR) (95% Cl)	Medication use *n* (%)	Adjustments
Wen et al. [30] 2021	1.33 [0.90, 1.97]^#^ 1.20 [0.71, 2.01]^b#^ 1.57 [0.77, 3.18]^c#^	RASI 416 (38); Corticosteroids 396 (36.1); Immunosuppressants 284 (25.9).	N.R.
Lu et al. [29] 2020	N.R.	N.R.	N.R.
Russo et al. [28] 2020	5.1 [1.12, 23.21]^c#^	RASI 93 (64); Corticosteroids or immunosuppressants 90 (62).	Age, sex, SBP, proteinuria/eGFR ratio and the presence of tubular atrophy and glomerulosclerosis.
Oh et al. [27] 2020	1.26 [1.19, 1.33]^#^ 1.19 [1.10, 1.28]^b#^ 1.38 [1.26, 1.51]^c#^	N.R.	Age, sex, SCr, DBP, anemia, BMI, T-cho, diabetes mellitus.
Ruan et al. [21] 2018	N.R.	RASI 125 (60.7); Corticosteroids 81 (39.3); Cyclophosphamide 25 (12.1).	N.R.
Liu et al. [19] 2018	1.97 [1.10, 3.54]^&^	RASI 603 (69.4); Immunosuppressants 641 (73.8).	Age, sex, chronic tonsillitis, hypertension, proteinuria, eGFR, serum IgA, serum C3, serum C4, Oxford classification, glomerulosclerosis, RASI and immunosuppressants.
Matsukuma et al. [20] 2017	1.97 [1.24, 3.11]*^&^	RASI (M group) 85 (47.4)^b^, 83 (39.2)^c^; RASI (H group) 103 (53.6)^b^, 136 (56.4)^c^; Corticosteroids (M group) 33 (18.4)^b^, 25 (11.8)^c^; Corticosteroids (H group) 28 (14.5)^b^, 44 (18.2)^c^	Age, sex, hypertension, BMI, proteinuria, eGFR, serum albumin, diabetes mellitus, diuretic use, allopurinol use and Oxford classification.
Caliskan et al. [23] 2016	1.29 [1.02, 1.62]^#^	RASI 59 (53.2); Only prednisolone 25(22.5); Prednisolone + immunosuppressant 14 (12.6); Immunosuppressant without prednisolone 13 (11.7).	Age, sex, MAP, eGFR, Hgb, SCr, albumin, proteinuria, hematuria, Oxford classification, intensity and pattern of staining for C3, C1Q, IgA, IgG, IgM.
Li et al. [18] 2016	2.27 [1.58, 3.27]^&^	RASI 1013 (90.4); Steroids 145 (12.9); Immunosuppressant 154 (13.7).	Proteinuria, hypertension, eGFR, hypoproteinemia, hypercholesterolemia and hypertriglyceridemia.
Moriyama et al. [25] 2014	1.24 [1.04, 1.48]^#^	RASI 292 (28.9); Corticosteroids 272 (26.9); Tonsillectomy plus steroids 118 (11.7).	Age, sex, BMI, MAP, eGFR, proteinuria, serum albumin total, T-cho, TG, U-RBC, IgA/C3 and Oxford classification.
Li et al. [17] 2014	1.09 [0.92, 1.3]^#^	RASI 676 (96.2); Glucocorticoids/immunosuppressant 316 (45.0)	Age, sex, Haas classification, eGFR, proteinuria, SBP and immunosuppressive therapy.
Cheng et al. [26] 2013	N.R.	RASI 255 (73.3); Corticosteroids 149 (42.8).	Age, sex, obese, smoke, hypertension, diabetes, RASI and corticosteroid treatment.
Shi et al. [22] 2012	N.R.	RASI 96 (27.2).	N.R.
Ohno et al. [24] 2001	N.R.	N.R.	N.R.

URR: unadjusted RR; ARR: adjusted RR; N.R.: not reported; PCS: prospective cohort study; RCS: retrospective cohort study; eGFR: estimated glomerular filtration rate; HPU: hyperuricemia; RASI: reninangiotensin system inhibitors; U-Prot: urinary protein excretion; U-RBC: urinary red blood count.

^&^RR: calculated by comparing hyperuricemia IgAN patients with non-hyperuricemia IgAN patients for the incidence of kidney failure events.

*RR calculated by comparing the H group (high SUA) with the M group (middle SUA) for the incidence of kidney failure events in IgAN patients.

^#^RR: calculated using uric acid level for the incidence of kidney failure events in IgAN patients.

^a^Median or mean.

^b^Male.

^c^Female.

Vague reporting of incomplete follow-up and dropout rates was the main limitation of most studies.

While hyperuricemia was defined by different cutoff levels in each study, most studies used a cutoff of SUA >7.0 mg/dL for males and SUA >6.0 mg/dL for females to define hyperuricemia.

### 3.2. The association between hyperuricemia and IgA nephropathy outcomes

**Figure 2.**
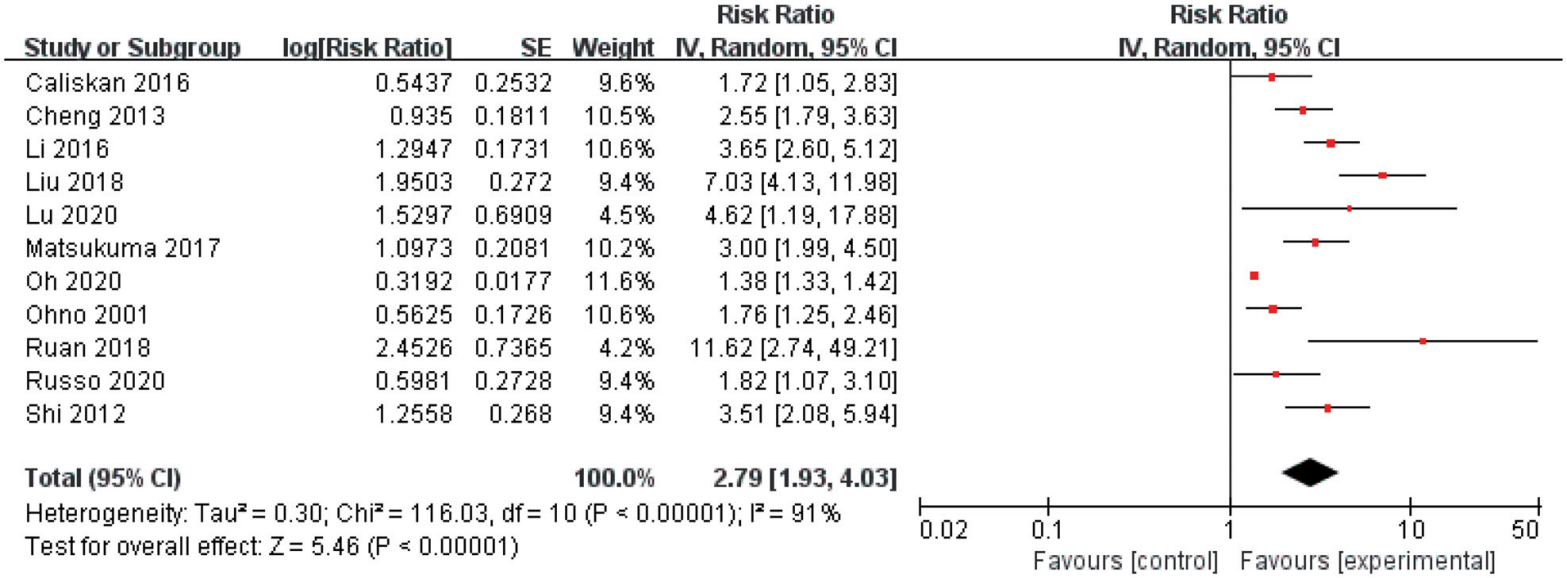
Forest plot of association between hyperuricemia and kidney failure events in IgAN (unadjusted RR).

Eleven of the 14 included studies calculated unadjusted RR to estimate the correlation between hyperuricemia and kidney failure events in IgAN [18–24,26–29]. In a random-effect model, our summary analysis showed a significant association between hyperuricemia and kidney failure events in IgAN (RR = 2.79, 95% CI = 1.93–4.03) with evidence of between-study heterogeneity (Chi^2^ = 116.03, *p* < 0.00001, *I^2^* = 91%) (Figure 2).

**Figure 3.**
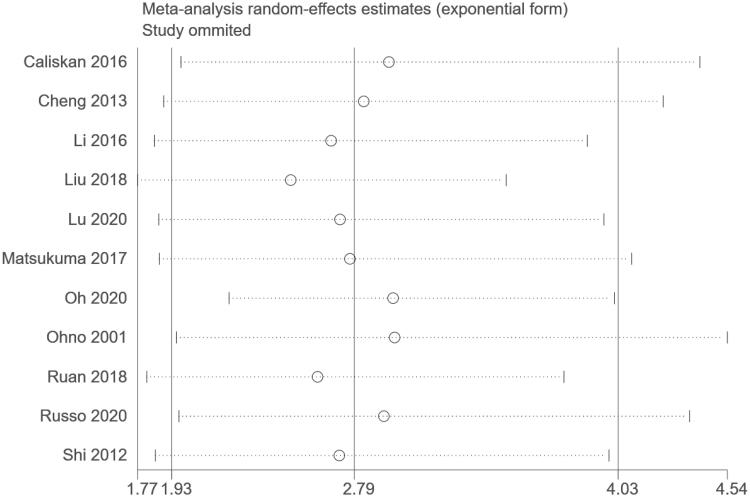
Sensitivity analyses.

A sensitivity analysis was conducted to explore potential sources of heterogeneity by excluding one study at a time and it confirmed the significant association between hyperuricemia and kidney failure events in IgAN (Figure 3). Subgroup analyses were also performed by study design, geographical region, definition of hyperuricemia, and duration of follow-up (Table 2). The association between hyperuricemia and kidney failure events in IgAN was consistent in all subgroups analyzed.

**Table 2.** Subgroup analyses of the relationship between hyperuricemia and kidney failure events in IgAN (unadjusted RR).

Subgroup	No. of studies	Pooled RR	95% Cl	Heterogeneity between studies
Study design				
Prospective	3	3.1	1.49–6.45	*p* = 0.0005, *I*^2^ = 87%
Retrospective	8	2.64	1.74–4.01	*p* < 0.00001, *I*^2^ = 90%
Geographical region				
Asian countries	9	3.14	2.02–4.87	*p* < 0.00001, *I*^2^ = 93%
Western countries	2	1.77	1.23–2.54	*p* = 0.88, *I*^2^ = 0%
Definition of hyperuricemia				
Reported	8	2.20	1.58–3.06	*p* < 0.00001, *I*^2^ = 82%
Not reported	3	4.1	2.65–6.35	*p* = 0.04, *I*^2^ = 69%
Follow-up				
>5 years	5	2.15	1.39–3.32	*p* < 0.00001, *I*^2^ = 92%
≤5 years	6	3.66	2.22–6.01	*p* = 0.002, *I*^2^ = 74%

**Figure 4.**
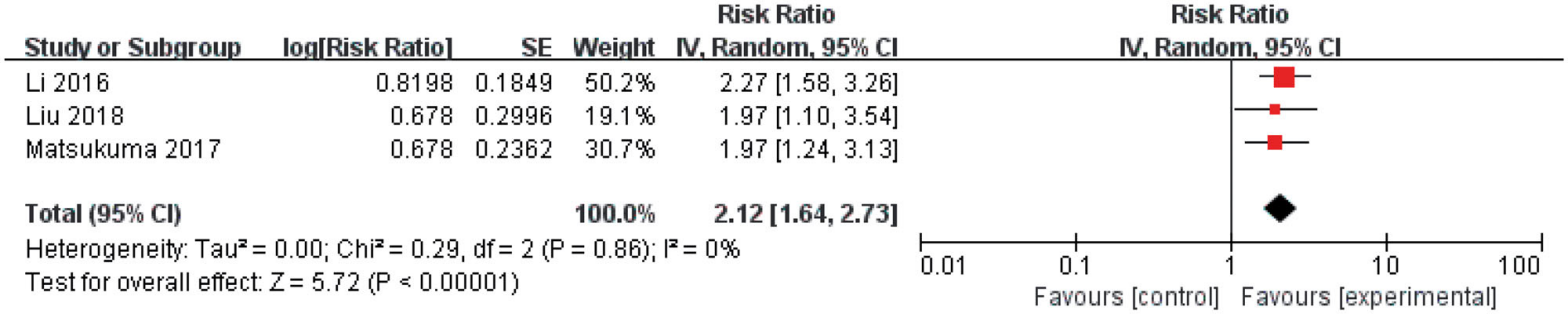
Forest plot of association between hyperuricemia and kidney failure events in IgAN (adjusted RR).

Of 11 studies that calculated unadjusted RR, three calculated the adjusted RR to estimate the correlation between hyperuricemia and kidney failure events in IgAN [18–20]. At least three potential confounders were adjusted for in these three studies. In a random-effect model, our summary analysis showed a significant association between hyperuricemia and kidney failure events in IgAN (RR = 2.12, 95% CI = 1.64–2.73). There was a very low heterogeneity between these three studies (Chi^2^ = 0.29, *p* = 0.86, *I^2^* = 0%) (Figure 4).

### 3.3. The association between SUA and IgA nephropathy outcomes

**Figure 5.**
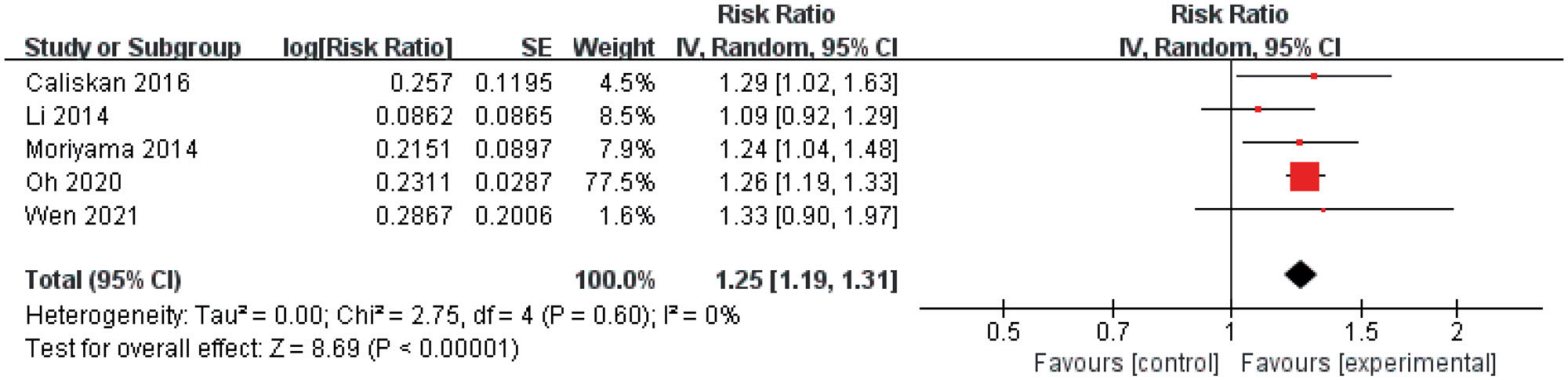
Forest plot of association between SUA level and the incidence of kidney failure events in IgAN.

Five of the 14 studies calculated an adjusted HR of increasing SUA by 1 mg/dL with regards to kidney failure events of IgAN [17,23,25,27,30]. At least three potential confounders were adjusted for in these five studies. A significant positive correlation between SUA and the incidence of kidney failure events in IgAN was observed in our random-effect model (RR = 1.25, 95% CI = 1.19–1.31). The heterogeneity of the three studies was negligible (Chi^2^ = 2.75, *p* = 0.6, *I^2^* = 0%) (Figure 5).

### 3.4. The association between SUA and IgAN-related kidney failure is consistent in males and females

**Figure 6.**
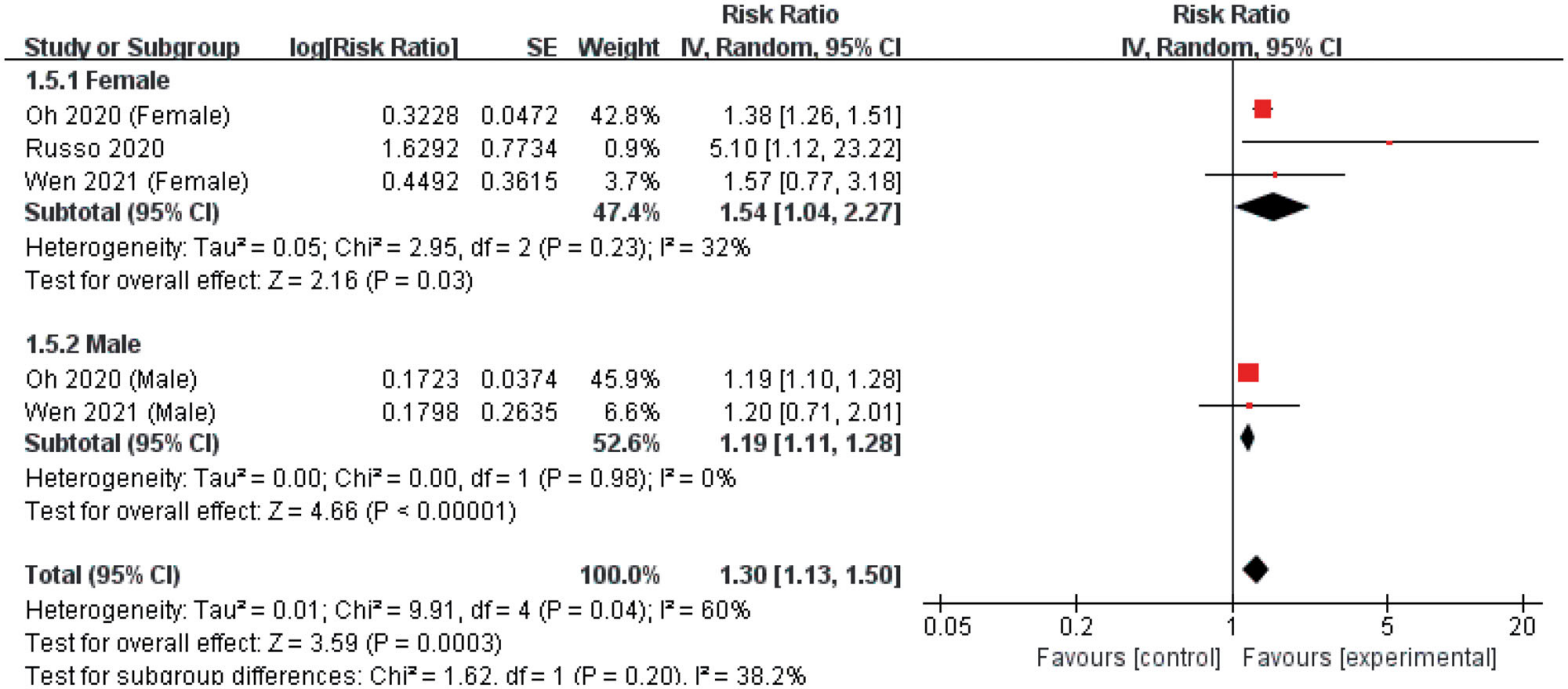
Forest plot of association between SUA level and the incidence of kidney failure events in IgAN according to sex.

A positive correlation was observed between SUA and the incidence of kidney failure events in IgAN for both females (RR = 1.54, 95% CI = 1.04–2.27, *I^2^* = 32%) and males (RR = 1.19, 95% CI = 1.11–1.28, *I^2^* = 0%). No difference was observed between female and male in the association between SUA and IgAN-caused kidney failure (test for subgroup difference, *p* = 0.20, *I^2^* = 38.2%) (Figure 6).

### 3.5. Publication bias

**Figure 7.**
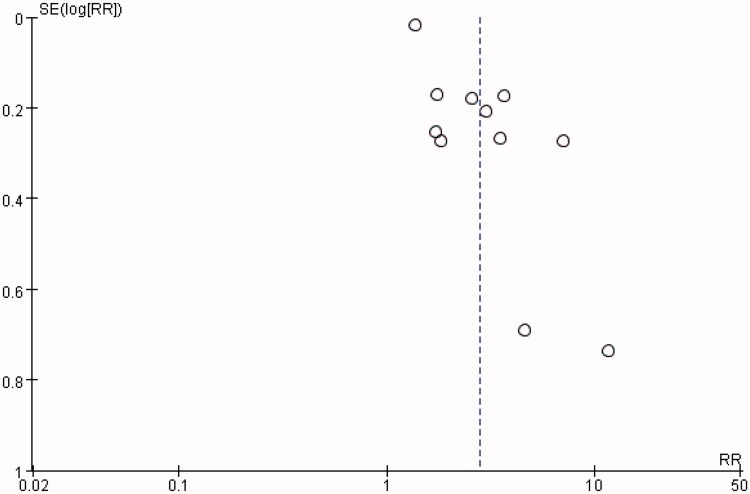
Funnel plot of association between hyperuricemia and kidney failure events in IgAN.

**Figure 8.**
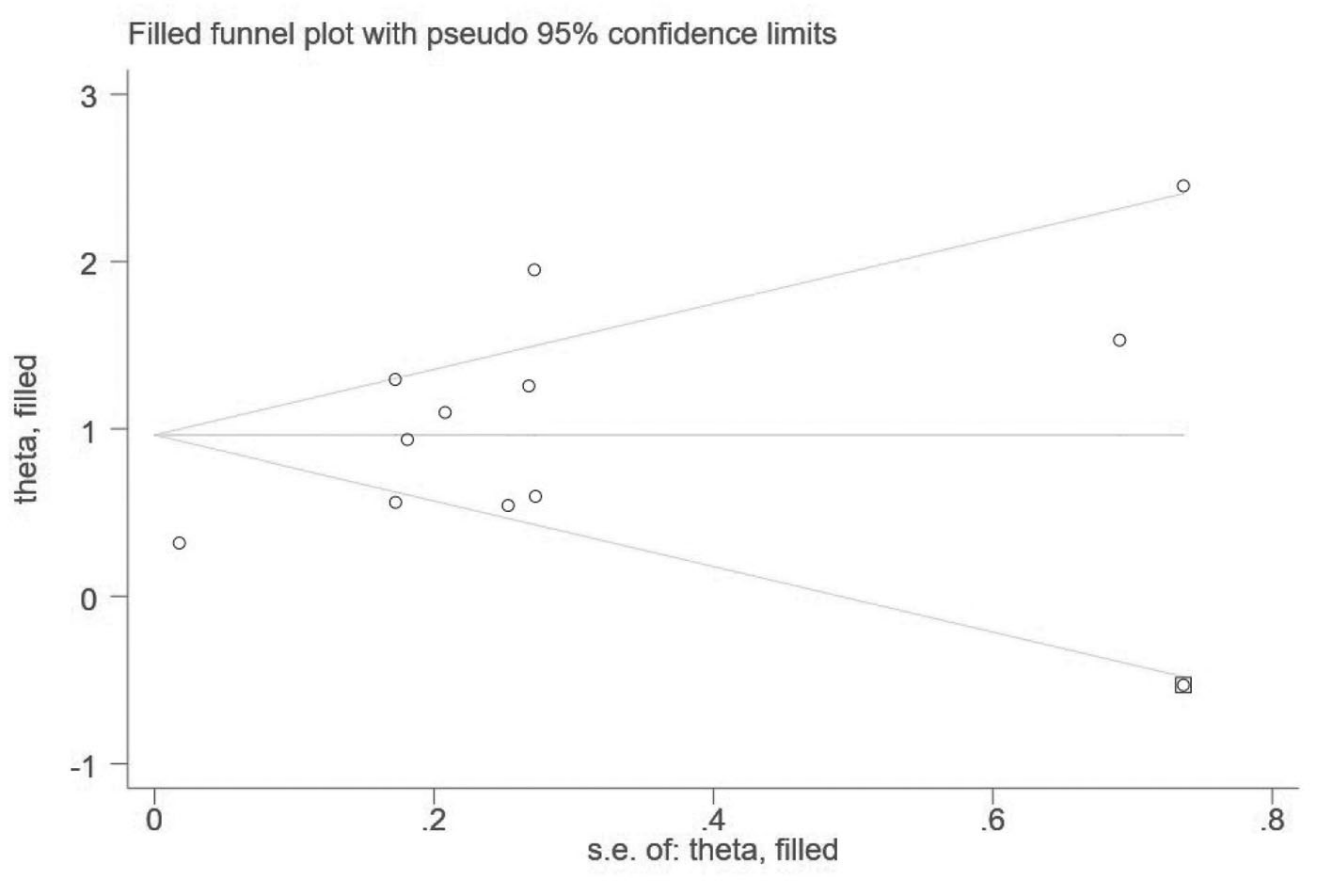
Trim-and-fill analysis estimating the number of possible missing studies for the association between hyperuricemia and kidney failure events in IgAN. The square represents the possible missing study.

Funnel plots suggested a publication bias for associations between hyperuricemia and kidney failure events in IgAN (Figure 7). Results of Begg’s test were negative (*p* = 0.755), however, Egger’s test indicated a significant publication bias (*p* = 0.001). Further analysis using the trim-and-fill test found that after estimating 1 missing study (Figure 8), the overall effect measure was an RR of 2.62 (95% CI, 1.83–3.75), which was slightly weaker than the originally reported overall effect measure.

No evidence of publication bias was found based on Begg’s (*p* = 0.806) or Egger’s (*p* = 0.688) tests for the association between SUA and the incidence of kidney failure events in IgAN.

## 4. Discussion

Several cohort studies have explored the risk factors for IgAN progression and identified massive proteinuria, impaired renal function, high blood pressure, persistent microhematuria, and certain types of renal pathology lesions as independent risk factors for IgAN progression [10,31,32]. Hyperuricemia is also common in individuals with IgAN. In fact, hyperuricemia IgAN patients presented more serious clinical and pathological features than those of individuals with lower SUA levels [21]. To date, hyperuricemia has attracted widespread attention as a potential risk factor in the occurrence and progression of CKD [33,34]. However, only a few studies have investigated the relationship between IgAN (a leading cause of CKD) and hyperuricemia.

SUA, which is mainly excreted in the urine through the kidney, is the result of purine metabolism. When SUA is beyond its physiological range, its excessive accumulation can result in crystal deposition in the kidney, thus damaging target organs. SUA levels can influence the pathophysiology of IgAN patients, and its effects on the kidney are mediated by a variety of biological phenomena. IgAN is a glomerular disease characterized by the deposition of IgA immune complexes in the glomerular mesangium, which induce the proliferation of glomerular mesangial cells [35]. SUA potentially contributes to glomerular damage through oxidative and pro-inflammatory effects in human mesangial cells [36] and induces the proliferation of mesangial cells in rats by activating the NADPH/ROS/ERk1/2 signaling pathway [37]. Recent studies have shown that hyperuricemia is essential to IgAN-associated tubule-interstitial lesions. Experiments *in vivo* and *in vitro* have shown that hyperuricemia may induce renal tubular injury mediated by epithelial-mesenchymal transition (EMT) through the activation of Wnt5a or the TLR4/NF-κB signaling pathway [38,39].

In addition, the pathological damage of the renal artery is a known complication of hyperuricemic patients. Uric acid can induce IgAN progression *via* its effect on arteriolar hyalinosis and intimal thickening [28]. Although the underlying mechanisms remain unclear, SUA levels play a crucial role in the progression of IgAN. A large-scale cohort study showed that SUA was positively correlated with IgAN progression, but the correlation was less conspicuous in patients with elder age, lower eGFR, or tubular atrophy/interstitial fibrosis [40]. Recently, another study indicated that elevated SUA was not an independent risk factor for low-eGFR in Chinese IgAN patients [41]. Thus, the prognostic value of SUA in IgAN patients remains controversial.

In our study, we calculated and pooled RRs before and after adjustment for relevant covariates to evaluate the relationship between hyperuricemia and IgAN-associated kidney failure. Results showed that IgAN patients with hyperuricemia were more likely to develop kidney failure compared with those without hyperuricemia in both unadjusted and fully adjusted models. Due to significant between-study heterogeneity in the unadjusted model (*I*^2^ = 91%), we used random-effect models to pool RRs and HRs. The subgroup and sensitivity analyses confirmed the stability of these results. The fully adjusted model showed no evidence of between-study heterogeneity (*I^2^*= 0%). Similarly, a significant positive correlation between SUA and the incidence of IgAN-related kidney failure was observed in the fully adjusted model with no evidence of between-study heterogeneity (*I*^2^ = 0%).

Previous studies have shown that IgAN varies largely in relation to geographic distribution [42]. Indeed, our subgroup analysis based on the geographic distribution of IgAN patients revealed a stronger correlation between hyperuricemia and IgAN-associated kidney failure in patients from Asian countries, compared with those from Western countries. This difference can be attributed to environmental and racial differences, but it may also be related to publication bias. Out of the 14 studies that were included in our meta-analysis, only two were conducted in Western countries (Turkey and Italy), while the remaining studies were conducted in Asian countries including China, Korea, and Japan. These geographic differences are consistent with a previous study that assessed the prevalence of IgAN upon a systematic review of 1619 publications throughout the world and found that IgAN is more common in Asians than it is in Caucasians [42]. Such difference may be due to a lower proportion of large-scale screening of urine in Western countries compared to those performed in Asian countries, or due to different criteria used by nephrologists for kidney biopsy. Therefore, the correlation observed in this subgroup analysis should be interpreted with caution, and more high-quality studies on the relationship between hyperuricemia and IgAN-associated kidney failure in Western countries are needed.

As hyperuricemia has different cutoff values for men and women, we performed a meta-analysis to detect whether the effect of SUA on IgAN progression varied according to sex. Our meta-analysis showed that elevated SUA was positively correlated with IgAN-caused kidney failure in both sex, but its effect was more pronounced in women than in men, which was consistent with previous studies [20,27,28,43]. Females seem to be more susceptible to uric acid-induced organ damage than males. Compared to male IgAN patients, SUA levels in female IgAN patients are related to more severe renal histopathological findings, such as mesangial matrix expansion, endocapillary proliferation, interstitial fibrosis, and tubular atrophy [44]. Indeed, estrogen plays an important role in reno-protection through the suppression of the urate reabsorptive transporter (URAT1) in the kidney, which results in an increase in uric acid excretion and decreased SUA levels [45]. Moreover, estrogen can negatively regulate TGF-ß synthesis; ovariectomy and estrogen deficiency accelerates the progression of glomerular injury [44]. Although these findings suggest a potential effect of estrogen, more high-quality researches are needed to clarify the underlying mechanisms of sex-specific effects of SUA levels in IgAN.

Despite the high quality of the included studies, our study had some limitations. First, for the relationship between hyperuricemia and IgAN-related kidney failure, only three studies provided adjusted data while eleven studies have used unadjusted data, which may decrease the strength of evidence of our findings. Second, visual inspection of the funnel plot and Egger’s test indicate potential risk of publication bias. Although further trim-and-fill test suggested that this publication bias did not impact the estimates, the potential risk of bias is inevitable. For example, heterogeneity of geographical region (mostly Asian countries), a different definition of hyperuricemia, and the primary outcome may all contribute to the potential risk of bias and limit the generalization of our findings. Third, a high heterogeneity (*I*^2^ = 91%) was found in our unadjusted model for the relationship between hyperuricemia and kidney failure events in IgAN. Given differences in study populations and baseline clinicopathological characteristics, it was not surprised to find significant heterogeneity in the unadjusted model. In addition, the subgroup and sensitivity analyses confirmed the stability of our results. Moreover, the fully adjusted models suggest that hyperuricemia and elevated SUA levels are independent prognostic factors of IgAN with no evidence of between-study heterogeneity (*I*^2^ = 0%).

Although hyperuricemia has been shown to be correlated with a poor prognosis in IgAN, whether urate-lowering therapy (ULT) has renoprotective effects has considerable controversies. Some studies believed that hyperuricemia had a detrimental impact on kidney function and advocated that ULT should be initiated in CKD patients when hyperuricemia was detected [46]. Conversely, a recent systematic review found there was insufficient evidence to support the renoprotective effects of ULT in hyperuricemia CKD patients [47]. Therefore, whether ULT could delay the progression of IgAN deserves further exploration. Non-pharmacological approaches, including exercise, weight loss, and low consumption of purine-rich food, fructose, and alcoholic beverages, can be recommended to all IgAN patients with hyperuricemia as adjunctive measures.

## 5. Conclusion

Our meta-analysis indicates that IgAN patients with elevated SUA levels have an increased risk of kidney failure events. These results suggest that hyperuricemia and elevated SUA levels are independent prognostic factors of IgAN patients. Therefore, high-quality randomized controlled trials are required to determine whether early prevention and timely control of SUA levels may delay kidney failure in patients with IgAN.

## Supplementary Material

Supplemental Material

Supplemental Material

Supplemental Material

Supplemental Material
