# Supplementary material for: Is hyperuricemia an independent prognostic factor for IgA nephropathy: a systematic review and meta-analysis of observational cohort studies
Source: Ren Fail. 2022 Feb 14;44(1):70–80. doi: 10.1080/0886022X.2021.2019589 (PMC8856039; doi:10.1080/0886022X.2021.2019589)
Supplement: Supplemental Material [file IRNF_A_2019589_SM2639.pdf]

Search strategies.

Pubmed:
#1 Hyperuricemia/
#2 (hyperuric* or uric acid)
#3 (#1 or #2)
#4 Glomerulonephritis,IGA/
#5 (Glomerulonephriti* IGA or Berger* disease or IGA nephropathy or Immunoglobulin A Nephropathy or IGA Type Nephritis)
#6 (#4 or #5)
#7 (#3 and #6)

Embase:
#1 Hyperuricemia/
#2 (hyperuric* or uric acid).mp. [mp=title, abstract, heading word, drug trade name, original title, device manufacturer, drug manufacturer, device trade name, keyword, floating subheading word, candidate term word]
#3 (#1 or #2)
#4 Glomerulonephritis, IGA/
#5 (Glomerulonephriti* IGA or Berger* disease or IGA nephropathy or Immunoglobulin A Nephropathy or IGA Type Nephritis).mp. [mp=title, abstract, heading word, drug trade name, original title, device manufacturer, drug manufacturer, device trade name, keyword, floating subheading word, candidate term word]
#6 (#4 or #5)
#7 (#3 and #6)

Central:
#1 Hyperuricemia/
#2 (hyperuric* or uric acid).mp. [mp=title, original title, abstract, mesh headings, heading words, keyword]
#3 (#1 or #2)
#4 Glomerulonephritis, IGA/
#5 (Glomerulonephriti* IGA or Berger* disease or IGA nephropathy or Immunoglobulin A Nephropathy or IGA Type Nephritis).mp. [mp=title, original title, abstract, mesh headings, heading words, keyword]
#6 (#4 or #5)
#7 (#3 and #6)

Open Grey:
(Glomerulonephritis, IGA OR Glomerulonephriti* IGA OR Berger* disease OR IGA nephropathy OR Immunoglobulin A Nephropathy OR IGA Type Nephritis) AND (Hyperuricemia OR hyperuric* OR uric acid)
