# Supplementary material for: Is hyperuricemia an independent prognostic factor for IgA nephropathy: a systematic review and meta-analysis of observational cohort studies
Source: Ren Fail. 2022 Feb 14;44(1):70–80. doi: 10.1080/0886022X.2021.2019589 (PMC8856039; doi:10.1080/0886022X.2021.2019589)
Supplement: Supplemental Material [file IRNF_A_2019589_SM2625.pdf]

The definition and method of measurement of the outcome of interest.

Author/year	Outcomes	Definition of outcomes	Formula to calculate eGFR	Method to measure Scr
**Wen et al. [30] 2021**	1) ESRD; or 2) 50% reduction in eGFR.	ESRD: 1) eGFR<15 ml/min/1.73 m2; or 2) initiation of dialysis.	CKD-EPI	N.R.
**Lu et al. [29] 2020**	1) initiation of dialysis; or 2) doubling of Scr.	N.R.	CKD-EPI	N.R.
**Russo et al. [28] 2020**	1) ESRD; or 2) all cause death.	ESRD: 1) start of chronic dialysis therapy; or 2) kidney transplantation.	CKD-EPI	Isotope-dilution mass spectrometry.
**Oh et al. [27] 2020**	IgAN progression.	IgAN progression: 1) 50% decline in the eGFR; or 2) the initiation of dialysis.	MDRD	Isotope-dilution mass spectrometry.
**Ruan et al. [21] 2018**	1) ESRD; or 2) doubling of Scr.	ESRD: 1) eGFR<15 ml/min/1.73 m2; or 2) initiation of dialysis or transplantation.	MDRD[#]	N.R.
**Liu et al. [19] 2018**	1) ESRD; or 2) eGFR decline ≥50%; or 3) death.	ESRD: 1) eGFR<15 ml/min/1.73 m2; or 2) initiation of renal replacement therapy, including haemodialysis, peritoneal dialysis or renal transplantation.	CKD-EPI	N.R.
**Matsukuma et al. [20] 2017**	ESRD.	ESRD: initiation of renal replacement therapy, including hemodialysis, peritoneal dialysis or kidney transplantation.	IDMS-MDRD	The Jaffe method or the enzymatic method. The Jaffe method were converted to values for the enzymatic method by subtracting 0.207 mg dl^{-1}.
**Caliskan et al. [23] 2016**	1) Kidney failure; or 2) eGFR decline ≥50%.	Kidney failure: eGFR <15 ml/min per 1.73 m2.	CKD-EPI	The enzymatic method.
**Li et al. [18] 2016**	1) ESRD; or 2) eGFR decline ≥50%.	ESRD: 1) eGFR＜15 ml/min/1.73 m2; or 2) initiation of dialysis or transplantation.	CKD-EPI	N.R.

**Moriyama et al. [25] 2014**	ESRD.	ESRD: 1) dialysis; or 2) renal transplantation.	IDMS-MDRD	N.R.
**Li et al. [17] 2014**	1) End stage kidney failure; or 2) eGFR halving.	N.R.	CKD-EPI	N.R.
**Cheng et al. [26] 2013**	ESRD.	ESRD: eGFR＜15 ml/min/1.73 m2.	MDRD$^{\#}$	N.R.
**Shi et al. [22] 2012**	1) eGFR decline ≥50%; or 2) the initiation of renal replacement therapy; 3) or death.	N.R.	MDRD$^{\#}$	The enzymatic method.
**Ohno et al. [24] 2001**	Doubling of Scr.	N.R.	N.R.	N.R.

eGFR: estimated glomerular filtration rate; Scr: serum creatinine; CKD-EPI: Chronic Kidney Disease Epidemiology Collaboration (CKD-EPI) equation[48]; MDRD: Modification of Diet in Renal Disease equation[49]; MDRD$^{\#}$: Modified MDRD equation for Chinese[50]; IDMS-MDRD: the modified isotope dilution mass spectorometry - modification of diet in renal disease for Japanese[51]; N.R. : not reported.
