# Supplementary material for: Is hyperuricemia an independent prognostic factor for IgA nephropathy: a systematic review and meta-analysis of observational cohort studies
Source: Ren Fail. 2022 Feb 14;44(1):70–80. doi: 10.1080/0886022X.2021.2019589 (PMC8856039; doi:10.1080/0886022X.2021.2019589)
Supplement: Supplemental Material [file IRNF_A_2019589_SM2617.pdf]

The NOS quality assessment of the included cohort studies

Study	Representativeness of the exposed cohort	Selection of the non exposed cohort	Ascertainment of exposure	Outcome of interest not present at start of study	Comparability of cohorts	Assessment of outcome	Follow-up long enough	Adequacy of follow up	Score
**Wen et al. [30] 2021**	YES	YES	YES	YES	NO	YES	YES	YES	7
**Lu et al. [29] 2020**	YES	YES	YES	YES	NO	YES	YES	NO	6
**Russo et al. [28] 2020**	YES	YES	YES	YES	NO	YES	YES	NO	6
**Oh et al. [27] 2020**	YES	YES	YES	YES	NO	YES	YES	YES	7
**Ruan et al. [21] 2018**	YES	YES	YES	YES	NO	YES	YES	NO	6
**Liu et al. [19] 2018**	YES	YES	NO	YES	YES	YES	YES	YES	7
**Matsukuma et al. [20] 2017**	YES	YES	YES	YES	YES	YES	YES	NO	7
**Caliskan et al. [23] 2016**	YES	YES	YES	YES	YES	YES	YES	NO	7
**Li et al. [18] 2016**	YES	YES	NO	YES	NO	YES	YES	NO	5
**Moriyama et al. [25] 2015**	YES	YES	YES	YES	YES	YES	YES	NO	7

**Li et al. [17] 2014**	YES	YES	NO	YES	YES	YES	YES	YES	7
**Cheng et al. [26] 2013**	YES	YES	YES	YES	NO	YES	YES	YES	7
**Shi et al. [22] 2012**	YES	YES	YES	YES	NO	YES	YES	NO	6
**Ohno et al. [24] 2001**	YES	YES	YES	YES	NO	YES	YES	NO	6

YES: the study meet the item in the scale; NO: the study didn't mentioned it (unclear) or failed to meet that item.
