# Supplementary material for: Is hyperuricemia an independent prognostic factor for IgA nephropathy: a systematic review and meta-analysis of observational cohort studies
Source: Ren Fail. 2022 Feb 14;44(1):70–80. doi: 10.1080/0886022X.2021.2019589 (PMC8856039; doi:10.1080/0886022X.2021.2019589)
Supplement: Supplemental Material [file IRNF_A_2019589_SM2605.pdf]

The MOOSE Checklist of the article

Criteria		Brief description of how the criteria were handled in the meta-analysis
**Reporting of background should include**		
√	Problem definition	Hyperuricemia has been reported to be correlated with IgA nephropathy. However, whether hyperuricemia or elevated serum uric acid (SUA) level is an independent prognostic factor of IgA nephropathy remained unknown.
√	Hypothesis statement	Hyperuricemia may be an independent prognostic factor for IgA nephropathy.
√	A statement of objectives that includes the study population, the condition of interest, the exposure or intervention, and the outcome(s) considered	Population: adult patients (> 18 years old) with biopsy-proven IgA nephropathy .<br>Condition of interest: observational cohort studies.<br>Exposures: hyperuricemia or an increase of 1 mg/dL in SUA levels.<br>Outcome: doubling of serum creatinine level, a two-fold reduction of eGFR level, ESRD or death.
**Reporting of search strategy should include**		
√	Qualifications of searchers	The credentials of the two investigators KZ and LT are indicated in the author list.
√	Search strategy, including time period included in the synthesis and keywords	Additional file 4 shows the search strategy of the study. Databases were systematically reviewed from inception to May 2021. Keywords list in the method part of article.
√	Databases and registries searched	PubMed, the Cochrane Central Register of Controlled Trials (CENTRAL), EMBASE and Open Grey were searched.
√	Search software used, name and version, including special features	We did not employ a search software. EndNote was used to merge retrieved citations and eliminate duplications.
√	Use of hand searching	We hand-searched bibliographies of retrieved papers for additional references.
√	List of citations located and those excluded, including justifications	Details of the literature search process are outlined in the flow chart. The citation list is available upon request.
√	Method of addressing articles published in languages other than English	Only studies published in English were included in our meta-analysis.
√	Method of handling abstracts and unpublished studies	Only published studies were included in our analysis.
√	Description of any contact with authors	No contact with authors.
**Reporting of methods should include**		

√	Description of relevance or appropriateness of studies assembled for assessing the hypothesis to be tested	Detailed inclusion and exclusion criteria were described in the methods section.
√	Rationale for the selection and coding of data	Data extracted from each of the studies were relevant to the population characteristics, study design, exposure, outcome, and possible confounding factors of the association. Two review authors will independently extract data. Any discrepancies that arise will be resolved by discussion, or by consulting a third author. A standardized form will be used to extract data from the included studies. This data will be assessed for quality, and then analyzed for synthesis of conclusions. If data from the selected studies is deemed to be missing, the appropriate study author will be contacted for clarification.
√	Assessment of confounding	The risk ratios between hyperuricemia and the kidney failure events of IgA nephropathy were evaluated before and after adjustment for known covariates. The risk ratios between elevated SUA level and the kidney failure events of IgA nephropathy were evaluated after adjustment for known covariates. All adjusted confounding factors were listed in Table 1. In the adjusted model, at least three potential confounders were adjusted for pooled-analysis.
√	Assessment of study quality, including blinding of quality assessors; stratification or regression on possible predictors of study results	Study quality were assessed by using New-Ottawa scale.<br>Subgroup analyses and sensitivity analysis were conducted.
√	Assessment of heterogeneity	Heterogeneity of the studies were explored within two types of study designs using Cochrane's Q test of heterogeneity and I^2 statistic that provides the relative amount of variance of the summary effect due to the between-study heterogeneity.
√	Description of statistical methods in sufficient detail to be replicated	Description of methods of meta-analyses, sensitivity analyses and assessment of publication bias are detailed in the methods.
√	Provision of appropriate tables and graphics	We included 1 flow chart detailing the terms used for database search and study selection, 3 forest plots for association between hyperuricemia and kidney failure events, SUA and kidney failure events in IgAN respectively, 1 forest plot for association between SUA and IgAN-caused kidney failure according to sex, 1 figure for sensitivity analysis, 1 table for the characteristics of the included

		studies, 1 table for subgroup analyses, 1 table for the definition and method of measurement of the outcome of interest, 1 funnel plot for publication bias and 1 funnel plot for trim-and-fill analysis.
**Reporting of results should include**		
√	Graph summarizing individual study estimates and overall estimate	Figure 2, 4, 5, 6.
√	Table giving descriptive information for each study included	Table 1.
√	Results of sensitivity testing	Detailed sensitive testing were described in results section (Figure 3, Table 2).
√	Indication of statistical uncertainty of findings	95% confidence intervals were presented with all summary estimates. Potential reasons for the observed heterogeneity were discussed.
**Reporting of discussion should include**		
√	Quantitative assessment of bias	The assessment of the publication bias was performed both visually inspected funnel plot and statistically tested for funnel asymmetry using the Begg's and Egger's regression tests. Moreover, the trim and fill methods were used to calibrate for publication bias.
√	Justification for exclusion	The exclusion criteria were described in method section. The process of exclusion were listed on Figure 1.
√	Assessment of quality of included studies	Study quality were assessed by using New-Ottawa scale.
**Reporting of conclusions should include**		
√	Consideration of alternative explanations for observed results	For IgA nephropathy patients with impaired renal function, hyperuricemia can be the result of decreased renal uric acid excretion, which could in turn further exacerbate kidney function. Therefore, the causal relationship between hyperuricemia and IgA nephropathy is far more complicated than a simple cause-and-effect relationship.
√	Generalization of the conclusions	Our meta-analysis indicates that IgAN patients with elevated SUA level increase the risk of kidney failure events during follow-up. Thus, early control of hyperuricemia may reduce IgAN-associated kidney failure. Non-pharmacological approaches, including exercise, weight loss and low consumption of purine-rich food, fructose and alcoholic beverages, can be recommended to all IgAN patients with hyperuricemia as adjunctive measures.
√	Guidelines for future research	High-quality randomized controlled trials are required to determine

		whether early prevention and timely control of SUA levels may delay kidney failure in patients with IgA nephropathy.
√	Disclosure of funding source	No separate funding was necessary for the undertaking of this systematic review.
